# Microarray analysis reveals genetic pathways modulated by tipifarnib in acute myeloid leukemia

**DOI:** 10.1186/1471-2407-4-56

**Published:** 2004-08-25

**Authors:** Mitch Raponi, Robert T Belly, Judith E Karp, Jeffrey E Lancet, David Atkins, Yixin Wang

**Affiliations:** 1 Veridex, LLC, a Johnson and Johnson company, San Diego, CA 9212, USA; 2 The Sidney Kimmel Cancer Center at Johns Hopkins, Baltimore, MD 21231, USA; 3 University of Rochester Cancer Center, Rochester, NY 14642, USA

## Abstract

**Background:**

Farnesyl protein transferase inhibitors (FTIs) were originally developed to inhibit oncogenic *ras*, however it is now clear that there are several other potential targets for this drug class. The FTI tipifarnib (ZARNESTRA™, R115777) has recently demonstrated clinical responses in adults with refractory and relapsed acute leukemias. This study was conducted to identify genetic markers and pathways that are regulated by tipifarnib in acute myeloid leukemia (AML).

**Methods:**

Tipifarnib-mediated gene expression changes in 3 AML cell lines and bone marrow samples from two patients with AML were analyzed on a cDNA microarray containing approximately 7000 human genes. Pathways associated with these expression changes were identified using the Ingenuity Pathway Analysis tool.

**Results:**

The expression analysis identified a common set of genes that were regulated by tipifarnib in three leukemic cell lines and in leukemic blast cells isolated from two patients who had been treated with tipifarnib. Association of modulated genes with biological functional groups identified several pathways affected by tipifarnib including cell signaling, cytoskeletal organization, immunity, and apoptosis. Gene expression changes were verified in a subset of genes using real time RT-PCR. Additionally, regulation of apoptotic genes was found to correlate with increased Annexin V staining in the THP-1 cell line but not in the HL-60 cell line.

**Conclusions:**

The genetic networks derived from these studies illuminate some of the biological pathways affected by FTI treatment while providing a proof of principle for identifying candidate genes that might be used as surrogate biomarkers of drug activity.

## Background

The investigative agent tipifarnib is a member of a new class of drugs that were designed to function as a non-peptidomimetic competitive farnesyltransferase inhibitor (FTI). The principal behind this drug class is that protein farnesylation is required for many cell-signaling processes and that dysregulation of cell signaling is thought to be instrumental in driving cell proliferation in several malignancies. The hypothesis that gave rise to this exciting class of drugs is that the inhibition of this enzyme would reduce the uncontrolled cell signaling and provide some control over cell division and malignant cell proliferation.

In hematological cancers, tipifarnib has shown significant inhibition of the proliferation of a variety of human tumor cell lines both *in vitro *and *in vivo *[1-3]. A recent phase I clinical trial of tipifarnib demonstrated a 32% response rate in patients with refractory or relapsed acute myeloid leukemia [4]. Furthermore, tipifarnib activity has also been seen in early clinical trials for patients with myelodysplastic syndrome (MDS) [5,6], multiple myeloma (MM) [7], and chronic myeloid leukemia (CML) [8].

Mechanism of action (MOA) and biomarker studies with tipifarnib have focused on the oncogenic Ras protein. However, it has since been shown that inhibition of Ras farnesylation does not account for all of the compound's actions. For example, FTIs do not require the presence of mutant Ras protein to produce anti-tumor effects [4]. Several other proteins have been implicated as downstream targets that mediate the anti-tumorigenic effects of FTIs. The regulation of RhoB, a small GTPase that acts down-stream of Ras and is involved in many cellular processes including cytoskeletal regulation and apoptosis, has been proposed as a mechanism of FTI-mediated anti-tumorogenesis [9]. Additional proteins involved in cytoskeletal organization are also known to be farnesylated including the centromere proteins, CENP-E and CENP-F, protein tyrosine phosphatase, and lamins A and B. Thus, one possible mode of action of FTI's may be due to their inhibiting effects on cellular reorganization and mitosis. In addition to possibly inhibiting cellular reorganization and mitotic pathways, it is also known that FTIs indirectly modulate several important signaling molecules including TGFβRII [10], MAPK/ERK [11], PI3K/AKT2 [12], Fas (CD95) and VEGF [13]. The regulation of these effectors can lead to the modulation of signaling pathways involving cell growth and proliferation, and apoptosis. Thus, FTIs may have complex inhibitory effects on a number of cellular events.

Where there are multiple candidate pharmacologic biomarkers as is the case with tipifarnib, a comprehensive, parallel study of all candidates is required. Here we describe the application of DNA microarray technology to the measurement of the steady-state mRNA level of thousands of genes simultaneously. This comprehensive experimental approach allows for the simultaneous analysis of candidate biomarkers as well as the generation of novel hypothesis on MOA and previously uncharacterized biomarkers. Biomarkers that enable the monitoring of drug response have the potential to facilitate clinical evaluation of the compound's safety and efficacy in humans. In the present paper we describe the use of global gene expression monitoring to identify genes and gene pathways that are modulated in acute myeloid leukemia (AML) following treatment with tipifarnib. Several genes involved in FTI biology were identified as being modulated following treatment with tipifarnib in addition to pathways involved with cytoskeletal organization, cell signaling, immunity, and apoptosis. This genome-wide approach of gene expression analysis has provided insight into genes that can be used as surrogate biomarkers for FTI drug activity as well as identifying putative pathways that are involved in the drug's anti-leukemic mechanism of action. This is the first successful report of the application of genomics to this novel class of drugs.

## Methods

### Cell culture

The AML cell lines AML-193, HL-60, THP-1, and U-937 were obtained from the American Type Culture Collection (Manassas, VA). Cells were grown in RPMI supplemented with 20% FBS. AML-193 was also supplemented with GM-CSF (10 ng/ml; PeproTech Inc., Rocky Hill, NJ), insulin (0.005 mg/ml; Sigma-Aldrich, St. Louis, MO), and transferrin (0.005 mg/ml; Sigma-Aldrich, St. Louis, MO). Cell numbers were counted in a hemocytometer and cell viability was determined by trypan blue dye exclusion assay. Tipifarnib was dissolved in 0.1% DMSO. The IC_50 _was defined as the dose at which the number of viable cells in the treated sample was 50% of that in the control. This was determined after 7 days of drug treatment. Cytotoxicity assays were performed in duplicate. Control cultures were grown in medium containing vehicle (0.1% DMSO) only. Cells were analyzed for apoptosis by treating with vehicle or tipifarnib (100 nM and 1 μM) over a 5-day time course. Cells were stained with Annexin V and propidium iodide daily according to the manufacturers protocol (Roche Applied Science, Indianapolis, IN) and analyzed by FACS.

### Bone marrow processing

Bone marrow samples were collected from consenting patients both before and during treatment with tipifarnib [4], diluted with PBS and centrifuged with Ficoll-diatrizoate (1.077 g/ml). White blood cells were washed twice with PBS, resuspended in FBS with 10% DMSO and immediately frozen at -80°C. Some characteristics of the two patient samples used in the present study are shown in Table 1.

### Ras mutational status

Analysis of activating mutations in N-*ras*, K-*ras*, *and *H-*ras *codons was determined by PCR and RFLP analysis as previously described [1].

### Microarray analysis

Total RNA was isolated using the Qiagen RNeasy kit (Qiagen, Valencia, CA) and treated with DNase1 (DNase1 kit, Qiagen, Valencia, CA) to remove any residual genomic DNA. Probe preparation was performed as previously described [14]. Linear amplification was performed on total RNA to obtain at least 15 μg of amplified RNA. Cell line mRNA and patient sample mRNA underwent one and two rounds of linear amplification respectively. Microarrays were generated and probes hybridized as described [15]. Samples were hybridized to arrays that contained 7452 cDNAs from the IMAGE consortium (Integrated Molecular Analysis of Genome and their Expression: ResGen™, Invitrogen Life Technologies, Carlsbad, CA) and Incyte libraries (Incyte, Palo Alto, CA). The intensity level of each microarray was scaled so that the 75^th ^percentile of the expression levels was equal across micro-arrays. To control for chip errors, replicate clones on each chip that displayed a coefficient of variance (CV) greater than 50% of the mean were excluded from the analysis. Since background intensity was a maximum of 30 relative fluorescent units (RFU) for all experiments, a threshold of 30 RFU was assigned to all clones exhibiting an expression level lower than this. The microarray data were then normalized by quantile normalization and logarithmically transformed before further analysis.

### Statistical analysis

Analysis of variance (ANOVA) and t-tests were used to investigate the effect of drug treatment and time and their interactions for each gene. Multiple hypotheses testing was controlled by applying the false discovery rate (FDR) algorithm [16]. All statistical analyses were performed in S-Plus 6.1 (Insightful Corporation). Ratio matrices were generated based on pair-wise analysis of treated versus control samples. Hierarchical clustering was performed using a correlation metric and complete linkage (OmniViz Pro™, OmniViz, Maynard, MA).

### Pathway analysis

A total of 1198 genes that had a false discovery rate (FDR) < 0.1 (p < 0.05) in at least one cell line were used for the pathway analysis. Gene refseq accession numbers were imported into the Ingenuity Pathway Analysis software (Ingenuity Systems). 898 of these genes were mapped to the Ingenuity database. Seventy-two of these genes were also affected in patient samples (p < 0.05, FDR < 0.3) and were, therefore considered to be significantly regulated by tipifarnib. The identified genes were mapped to genetic networks available in the Ingenuity database and were then ranked by score. The score is the probability that a collection of genes equal to or greater than the number in a network could be achieved by chance alone. A score of 3 indicates that there is a 1/1000 chance that the focus genes are in a network due to random chance. Therefore, scores of 3 or higher have a 99.9% confidence of not being generated by random chance alone. This score was used as the cut-off for identifying gene networks significantly affected by tipifarnib.

### Real Time RT-PCR

The genes and primers used for RT-PCR are listed in Table 2. Due to the limited amount total RNA from the patient samples, RNA that had been through one round of linear amplification was used. The Roche Molecular LightCycler (Roche Applied Science, Indianapolis, IN) with Syber Green I system detection was used for real time PCR. PCR thermocycling consisted of denaturation at 95°C for 45 seconds, followed by 30 cycles at 62°C for 10 seconds, and 72°C for 12 seconds. Samples were run in triplicate with both test primer sets and the control gene eukaryotic elongation factor 1 alpha (EEF1A1). This gene was used to control for differences in the amount of target material since initial microarray experiments found that expression of the EEF1A1 gene did not vary significantly between drug-treated and control cells. A standard curve was also run in each PCR reaction. Fold changes were calculated by normalizing the test crossing thresholds (Ct) with the EEF1A1 amplified control Ct.

## Results and Discussion

### Response of AML-like cell lines to tipifarnib

Tipifarnib inhibited the growth of 4 human AML cell lines in a dose-dependent manner. The IC_50 _of these cell lines when treated with tipifarnib ranged from 19 to 134 nM (Table 3). The mutation status of the *ras *oncogenes in the AML cell lines are also shown. These data indicate that the four AML-like cell lines are sensitive to tipifarnib treatment at concentrations well below the micromolar concentrations that is achievable in the bone marrow of leukemia patients [4]. However, there was no correlation between the type of *ras *mutation and sensitivity to the drug. These data are consistent with the activity of tipifarnib *in vivo *and allowed for further characterization of gene expression changes in these cells after treatment with pharmacologically relevant drug concentrations.

### Identification of genes differentially expressed in tipifarnib-treated AML cells

**Figure 1:**
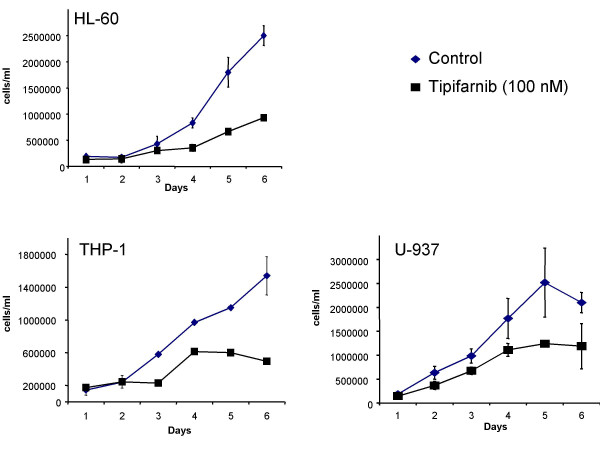
Growth profiles of AML cell lines treated with tipifarnib. Duplicate cultures were inoculated into 6-well plates at an initial concentration of 1 × 10^5^cells/ml. Tipifarnib was supplemented at a concentration of 100 nM in 0.1% DMSO. Duplicate control cultures were grown in medium containing 0.1% DMSO only. Duplicate cultures were harvested daily for a total of six days. Error bars are standard deviations.

**Figure 2:**
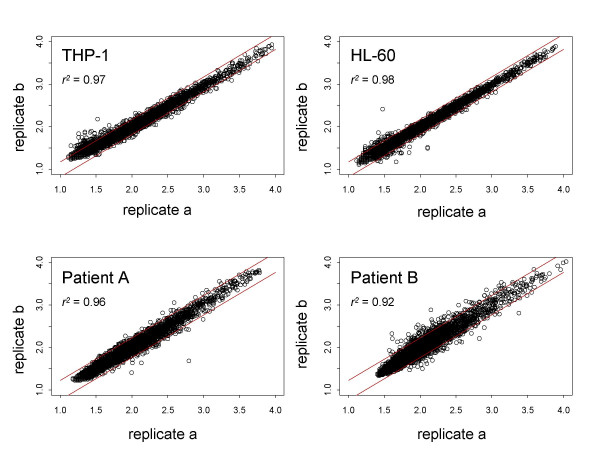
Scatter plot analysis of microarray data. Duplicate THP-1 and HL-60 cell line cultures were harvested, processed independently, and hybridized to the cDNA array. Duplicate samples from patients A and B were also analyzed for reproducibility. The lines on the scatter plots indicate the 1.5-fold and 1.7-fold boundaries used for selecting genes with differential expression in cell line samples and patient samples, respectively. Less than 5% of genes were outside these fold-change thresholds. Linear regression was performed and correlation coefficients are shown. Axes show the fluorescence intensity associated with each gene (log_10_).

**Figure 3:**
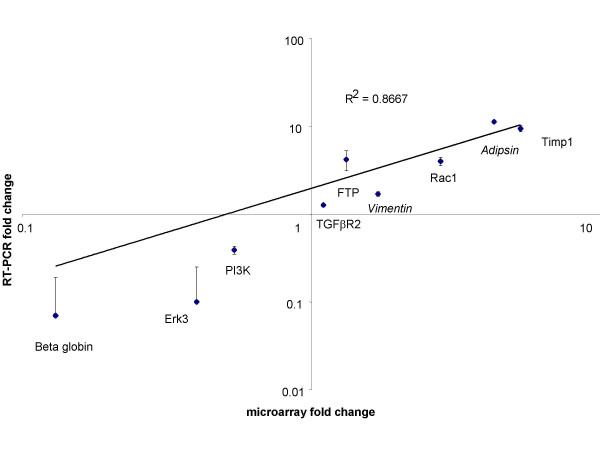
Real time RT-PCR validation of microarray data. Nine genes were randomly selected for real time RT-PCR. Two of these genes (adipsin and vimentin) were identified as being significantly regulated in both the cell lines and in *de novo *AML patients. The "fold-change" (Log_2_) of the RNA transcripts was calculated in the patient who responded to tipifarnib (patient A) by using the treated (day 15) versus the matched baseline sample for both PCR and microarray data. Linear regression analysis was performed and the coefficient of variation was calculated. Italicized genes were identified as being significantly regulated by tipifarnib in both AML cell lines and patient samples. Error bars are standard deviations.

We next asked what genes are modulated following treatment of AML cells with tipifarnib and if there are differences between the affected gene networks in cell lines compared to primary cells from patients. To this end we first selected the three most sensitive cell lines and treated them with tipifarnib or vehicle alone over a 6-day time course. A standard concentration of 100 nM tipifarnib was chosen to ensure exposure within the pharmacologically active range of the compound (Fig 1). Samples for RNA analysis were harvested daily from duplicate cell cultures. Message RNA was isolated, amplified and hybridized to the cDNA microarrays containing approximately 7000 genes. Based on scatter plot analysis the microarray data was found to be highly reproducible between duplicate samples (Fig 2). A one-way ANOVA was employed to identify genes that were significantly changed over the 6-day time course compared to time-matched controls. A total of 1198 genes were significantly regulated (p < 0.05 with a false discovery rate of less than 10%) in at least one of the cell lines over the time course (Supplementary Figure A [see Additional file 1]). We also had access to bone marrow samples from two newly diagnosed AML patients enrolled in a phase I trial for tipifarnib [4]. The gene expression profiles in pre-treated leukemia cells were compared to those during drug treatment at days 8, 15 and 22. 1016 genes were significantly changed (p < 0.05, FDR < 0.3) during farnesyltransferase inhibition in vivo (Supplementary Figure B [see Additional file 1]). A total of 180 genes were common between the cell line and patient data sets, 141 of these had known functions (see Additional file 2). Real time RT-PCR showed good agreement with the microarray data (r^2 ^= 0.87; Fig 3).

There are several known targets of FTIs including ras, RhoB, centromere proteins, lamins, PI3K/AKT, and TGFβRII [3,10]. While the majority of these genes were present on our expression array (except the lamins) we only found k-ras to be significantly regulated. However, while not significant, up-regulation of TGFβRII was confirmed by RT-PCR (Fig 3). The absence of strong regulation of TGFβRII in the current data set may be due to the different FTI and/or the different culture conditions that were employed compared to previous reports [10]. Interestingly, k-ras was significantly down-regulated in our system. While k-ras is a target of FTIs it has been shown to undergo alternative geranylgeranylation when farnesylation is inhibited and may therefore not be an important anti-tumorgenic target post-translationally; however, it maybe a relevant target at the transcriptional level [17]. Repression of k-ras transcription has also been shown recently in a mouse model designed to identify genes that are related to the transformation-selective apoptotic program triggered by FTIs [18]. K-ras may therefore warrant further investigation as a candidate transcriptional target of FTIs.

### Identification of genetic networks affected by tipifarnib

To further refine the list of FTI-affected genes we next investigated which of these genes are known to interact biologically. To this end we carried out pathway analysis on the above 180 genes using the Ingenuity Pathway Analysis (IPA) tool. Seventy-nine (72 unique) of these 180 genes mapped to genetic networks as defined by the IPA tool. These networks describe functional relationships between gene products based on known interactions in the literature. The tool then associates these networks with known biological pathways. Five networks were found to be highly significant in that they had more of the identified genes present than would be expected by chance (Table 3). These networks were associated with the cell cycle, apoptosis, proliferation, chemotaxis, and immunity pathways. The study by Kamasani et al also found cell cycle pathways were repressed and immunity and cell adhesion pathways were activated by FTI treatment [18].

**Figure 4:**
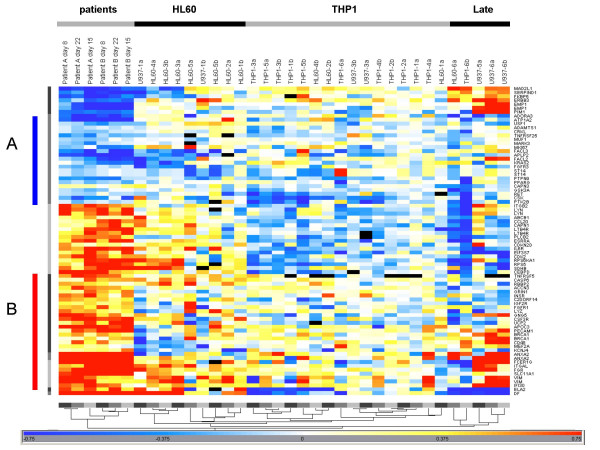
Hierarchical clustering of genes regulated after tipifarnib treatment. A fold-change ratio was calculated using the treated sample and its matched untreated sample. Duplicate samples are indicated with suffices "a" and "b". Number suffices indicate day of treatment. The color bar indicates the fold-change (log_2_). Red is up-regulated, blue is down-regulated. White indicates no change. Cluster A and B were associated with genes that were largely down- and up-regulated across all samples, respectively.

The 79 genes were then analyzed by two-way hierarchical clustering to compare the expression profiles of the AML samples (Fig 4). A number of observations could be made using this visual approach. First, although there were some outliers, the majority of duplicate samples clustered close together again demonstrating the reproducibility of the results. Similarly, a number of replicate clones of the same gene clustered next to each other thereby improving the confidence of the microarray data. As expected, samples from the same cell line or patient clustered together. However, samples from late in the time courses have very different expression profiles possibly reflecting greater differences in the transcriptional activity between control and treated cells at this late stage of drug treatment. Interestingly, the cluster analysis showed that the HL-60 profile was most similar to the patient samples indicating it has a more similar response to tipifarnib compared to the patient cells than THP-1 and U-937. This similarity cannot be associated with FAB sub-type since HL-60 was isolated from a patient with M2 AML and the patients examined in this study were M4 and M5 sub-types. Therefore, it is suggested that the different expression profiles seen are due to other genetic differences that impact the specific down-stream effects of FTI inhibition. This may be important when considering appropriate models for FTI investigations.

While the cell lines portrayed higher heterogeneity in expression changes compared with the patient samples, the hierarchical clustering did reveal a common set of up- and down-regulated genes. A set of 23 genes was found to be down-regulated in the cell line and patient samples (Fig. 4). The major network associated with these genes contained several involved in proliferation including CSK, FGFR3, KRAS2, PPARG, RET, and USF1. Alternatively, 29 genes were commonly up-regulated and network analysis of these revealed activation of apoptotic- and immune-related genes, including CASP6, CD48, FGR, IGF2R, PECAM1, and TNFRSF5. It will be of interest to investigate these genes further to see if they are transcriptional targets of FTIs and if their regulation is additive or synergistic to FTI efficacy.

**Figure 5:**
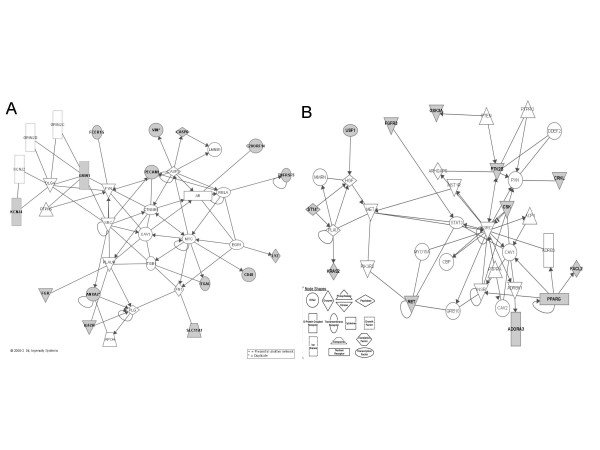
Networks of genes commonly regulated after tipifarnib treatment. **(A) **Twenty-three genes that were down-regulated in patient leukemic cells and AML cell lines were analyzed by the Ingenuity Pathway Analysis tool. The shown major network that was found to be significantly down-regulated by tipifarnib was associated with proliferation (p = 10^-10^). **(B) **Twenty-nine genes that were up-regulated were also analyzed for associated networks that were significantly affected by tipifarnib. The shown network was significantly associated with apoptosis (p = 10^-10^) and immunity (p = 10^-7^). Shaded genes are the genes identified by microarray analysis and others are those associated with the regulated genes based on the pathway analysis. The meaning of the node shapes is also indicated. Asterisks indicate genes that were identified multiple times.

Due to the stringency of our gene selection process it is likely that many genes that are indeed regulated by FTIs, were not identified. For instance, as noted above, of the targets known to be affected by FTIs we identified only k-ras at the transcriptional level. However, the use of pathway analysis tools allows for the identification of networks of genes that are known to interact with each other. This procedure therefore provides additional confidence in the selected genes as well as clues to other genes that may also be regulated but not identified as being significant by the microarray analysis. For example, the network of up-regulated genes (Fig 5A) includes the lamin B gene, which is indeed a direct target of FTIs. Also, the PIK3R2 gene, which regulates AKT and is a known target of FTIs [3], can be found in the down-regulated network of genes (Fig. 5B). This illustrates that the pathway analyses correctly identifies genes that have previously been demonstrated to be either direct or indirect targets of farnesyltransferase inhibition and provides a greater context for screening candidate genes modulated by FTIs.

### Investigation of apoptosis

**Figure 6:**
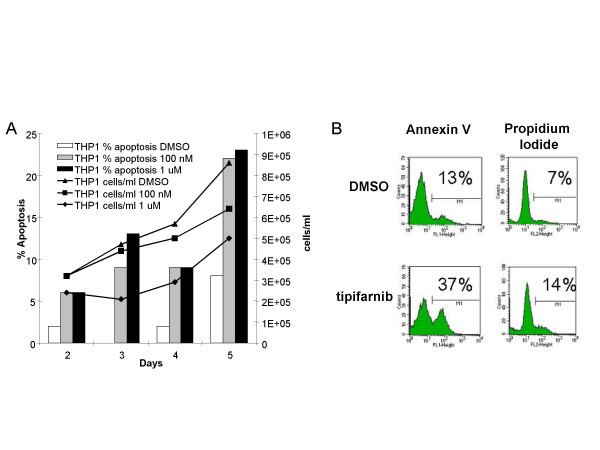
Detection of tipifarnib-mediated apoptosis in AML cells. **(A) **Annexin V staining shows that a decrease in cellular proliferation correlates with an increase in apoptosis in the THP1 cell line following treatment with tipifarnib. **(B) **Apoptosis assay of control and treated cells at day 5. Annexin V stains both apoptotic and necrotic cells, propidium iodide stains necrotic cells only.

Since a number of apoptotic genes were identified as being affected by tipifarnib we performed experiments in THP-1 and HL-60 cell lines to verify if they were indeed undergoing apoptosis. Previous reports have shown that two other FTIs can induce apoptosis in myeloid leukemia cell lines [11] and that tipifarnib causes apoptosis in other malignancies including multiple myeloma [19], and melanoma [1]. Annexin V staining demonstrated a significant increase in FTI-mediated apoptosis in THP-1 for both 100 nM (p = 0.027) and 1 uM (p = 0.032) concentrations of tipifarnib (Fig 6). A maximum of 23% apoptotic cells were demonstrated at day 5 (Fig. 6). No difference in the level of apoptosis was seen between 100 nM and 1 μM of tipifarnib. While apoptosis was activated in the HL-60 cell line this was found to be non-specific since control cells also exhibited this phenomenon during cell culture (data not shown). The lack of FTI-specific apoptosis in HL-60 is consistent with a recent report that also failed to demonstrate tipifarnib-mediated apoptosis in primary AML blasts [20]. However, in that report apoptosis was measured only two days after treatment where here we found a marked increase in apoptosis at days 3–5. Therefore, our data indicate that tipifarnib can cause apoptosis in AML but may not be detectable at early time points or in AML with certain genetic backgrounds.

## Conclusions

Tipifarnib is one of three FTIs that are currently in clinical trials for treating a variety of cancers [21] and it is showing promise in hematological malignancies [3-8]. While FTIs were originally designed to inhibit the function of the *ras *oncogene it has been recently demonstrated that there is no correlation between patient response and *ras *mutational status [4]. Additionally, it is clear that other targets of FTIs exist that provide equally important anti-cancer properties. We have reported the use of microarray analysis of both primary human AML cells and AML cell lines following treatment with tipifarnib in order to identify genes and gene pathways that are modulated by this FTI. In particular, genes involved in signaling pathways, down-stream cytoskeletal pathways, and apoptotic events were described. Pharmacodynamic markers that are currently used in the clinic, such as lamin A and HDJ2 [22], are direct markers of farnesyltransferase inhibition while the majority of genes identified in this work are likely downstream transcriptional targets. Both of these current candidate markers were not present on our microarrays so we did not report on their expression changes. Further analysis will be required to elucidate whether the expression changes seen in our work are due to direct or indirect effects of FTIs. Also, while the currently used clinical biomarkers do not correlate with patient response to FTIs the genes identified here may be candidates for patient stratification [3]. We are therefore in the process of examining bone marrow specimens from larger phase 2 clinical trials with the aim of validating the panel of pharmacodynamic gene expression markers we have identified here. Such pharmacogenomic analysis will be very important in further elucidating the action of FTIs while providing a platform for identifying patients who could potentially respond to tipifarnib therapy.

## Competing interests

MR, RTB, DA, and YW are employees of Veridex LLC., a Johnson and Johnson company. JEK and JEL have received fees from Johnson & Johnson Pharmaceutical Research and Development in the last 5 years.

## Author's contributions

MR wrote the manuscript and performed experiments. RTB performed Ras analysis. DA helped conceive the experiments. JEK and JEL were principal investigators in the clinical trials from which samples were received. YW helped conceive the experiments. All authors read and approved the final manuscript.

## List of abbreviations

AML: acute myeloid leukemia; FDR: false discovery rate; FTI: farnesyltransferase inhibitor; MDS: myelodysplastic syndrome; MOA: mechanism of action; MM: multiple myeloma; CML: chronic myeloid leukemia

## Pre-publication history

The pre-publication history for this paper can be accessed here:



## Supplementary Material

Additional File 1 A. Two-way hierarchical clustering of 1198 genes regulated in three AML cell lines after tipifarnib treatment. A fold-change ratio was calculated using the treated sample and its matched untreated sample. B. Gene expression changes in patient AML cells. Two-way hierarchical clustering of 1016 genes regulated in two AML patients over a three-week time course. Click here for file

Additional File 2 A list of 141 genes with known function that were regulated in both the cell line and patient data sets. Click here for file

## Figures and Tables

**Table 1:** Characteristics of patient AML samples.

Patient	Dose (mg)	Age/sex	Diagnosis	Stage	Antigen expression	*ras *status
B	100	59 M	M5, de novo AML	Relapse	CD34+, CD33+	WT
A	300	75 M	M4, de novo AML	New	CD34+, CD33+	WT

WT = wild-type

**Table 2:** Primer sequences used for RT-PCR.

**Gene**	**Forward Sequence**	**Reverse Sequence**
RAC1	CACGATCGAGAAACTGAAGGA	AGCAGGCATTTTCTCTTCCTC
TIMP1	TACTTCCACAGGTCCCACAAC	GTTTGCAGGGGATGGATAAAC
TGFβR II	CAGCGTTTCAAAAAGTGAAGC	CTAGACTGGGGTCCAGGTAGG
βGLOGIN	GCAACCTCAGACAGACACCAT	ACCTTAGGGTTGCCCATAACA
PI3K	TGAGCAAGAGGCTTTGGAGTA	TTCCTATGCAATCGGTCTTTG
ERK3	GAGCCAGTAGAGGATGGGAAG	GATGAGGAATTTGAGGGGAAG
VIMENTIN	ATCGATGTGGATGTTTCCAAG	TGTCTCCGGTACTCAGTGGAC
FTP	ATCCCTTAGCATCAGCTCCTC	CGTTCTTTTGGCATTAGTTGG
ADIPSIN	CCTGCATCTGGTTGGTCTTTA	AGCCTCCTGAGTAGCTGGAAC
EEF1A1	GATGCATTGTTATCATTAACC	CATGCAAGTTTGCTGAGCTG

**Table 3:** Anti-proliferative effects of tipifarnib for AML cell lines.

Cell line	IC_50 _(nM)†	*ras *status
AML-193	134	H-*ras*(12), N-*ras*(13)
HL-60	24	H-*ras*(12)
THP-1	19	K-*ras*(13) N-*ras*(12, 61)
U-937	44	Wild-type

† The IC_50 _was calculated from two independent experiments. The mean value is shown.

**Table 4:** Genetic networks affected by tipifarnib

**Network**	Genes in Ingenuity network*	**Associated pathways**	**Score†**
1	CD226, CD24, CD36, CD63, **CEBPD**, **CRKL**, CSF2RB, **CSK**, **DF**, DOK1, **FACL2**, FCAR, **FCER1G**, **FGFR1**, GAB2, GP6, **GRIN1**, ICAM1, ICAM2, ICAM3, INPP5D, **INSR**, **ITGAL, ITGB2**, LAT, **LYN**, **MAD2L1**, NEDD9, **PECAM1**, **PPARG**, **PTK2B**, SORBS1, STAT5B, SYK, **UCP2**	Immunity Inflammation Apoptosis Cell death Adhesion	24
2	**ABCB1**, **ADORA3**, **APOC3**, AR, **BRCA1**, **C20ORF14**, CAV1, CCNA1, CCNB1-RS1, CCND1, **CDK2**, CDKN2A, **CDKN2D**, CKS1B, CREM, E2F1, E2F4, **ELA2**, **ESRRA**, **FGFR3**, GNAQ, GNB1, **GNG5**, HDAC1, ICAM1, JUN, KLKB1, LAMP1, **MKI67**, **PLCB2**, PTPN2, **PTPN9**, RB1, **RBBP2**, TTK	Cell cycle Transcription Apoptosis	20
3	ABCB1B, ARL6IP, BAX, BCL2, **CAPN1**, CDC25C, CDKN1A, CEBPE, CREM, CSF2RA, **CSF3R**, CTCF, **EMP1**, F2, **FACL3**, GPI, **LTB4R**, MAPK14, **MARK3**, **MEF2A**, MYC, MYCN, **PIM1**, PPP1R15A, PTGS2, RPL21, RPL6, RPS12, RPS16, **RPS5**, **SERPIND1**, SLC19A1, SP1, TP53, **USF1**	Cell cycle Cell death Proliferation	13
4	ANXA1, **ANXA2**, APOH, BCR (BREAKPOINT CLUSTER REGION), CD22, **CD48**, DOK2, EIF3S2, EIF3S6, **EIF3S7**, EPHA4, **ERBB3**, **FGR**, FN1, FYN, GRB2, HCLS1, **IGF2R**, **IL6R**, IL6ST, **KRAS2**, LCK, LIF, LY6, PLAU, PLAUR, PLG, **RET**, S100A10, SHC1, **SLC11A1**, **ST14**, TNC, TRPV4, ZFP106	Chemotaxis Proliferation Apoptosis	13
5	ACT1, **APLP2**, BIRC4, CAMKL, CASP3, **CASP6**, CASP8, CASP9, **CCL20**, DRPLA, EGR1, EIF4B, **FKBP5**, HNF4A, **IFI30**, LMNB1, **LYZ**, NR0B2, NR3C1, NUMA1, PCYT1A, PLEC1, PRKCZ, RELA, RELB, RIPK2, **RPS6KA1**, TAX1BP1, **TNFRSF25**, **TNFRSF5**, TRAF1, TRAF3, TRAF5, **VIM**, WEE1	Apoptosis Cell death Proliferation	11

* Bold genes are those identified by the microarray analysis. Other genes were either not on the expression array or not significantly regulated. † A score of > 3 was considered significant (p < 0.001).
