# Supplementary material for: Microarray analysis reveals genetic pathways modulated by tipifarnib in acute myeloid leukemia
Source: BMC Cancer. 2004 Aug 25;4:56. doi: 10.1186/1471-2407-4-56 (PMC516036; doi:10.1186/1471-2407-4-56)
Supplement: Additional File 1 — A. Two-way hierarchical clustering of 1198 genes regulated in three AML cell lines after tipifarnib treatment. A fold-change ratio was calculated using the treated sample and its matched untreated sample. B. Gene expression changes in patient AML cells. Two-way hierarchical clustering of 1016 genes regulated in two AML patients over a three-week time course. [file 1471-2407-4-56-S1.doc]

# Supplementary Figure

**Supplementary Figure**. **A.** Gene expression changes in AML cell lines. Two-way hierarchical clustering of 1198 genes regulated in three AML cell lines after tipifarnib treatment. A fold-change ratio was calculated using the treated sample and its matched untreated sample. Duplicate samples are indicated with suffices “a” and “b”. Number suffices indicate day of treatment. Red is up-regulated, blue is down-regulated. White indicates no change. Clusters I and II (boxed) show genes that are largely up-regulated in at least two cell lines. Cluster III (boxed) shows genes that are largely down-regulated in at least two cell lines. These three, boxed clusters make up over half of the 1198 affected genes. **B.** Gene expression changes in patient AML cells. Two-way hierarchical clustering of 1016 genes regulated in two AML patients over a three-week time course. A fold-change ratio was calculated using each time course sample and its matched sample prior to patient treatment. Number suffices indicate day of treatment. Red is up-regulated, blue is down-regulated. White indicates no change.
